# Editorial: Innate immune response and interaction with adaptive immunity: role in tuberculosis immunology/vaccinology

**DOI:** 10.3389/fimmu.2025.1609680

**Published:** 2025-05-23

**Authors:** Elisa M. Rouse Salcido, Elena Stylianou, Styliani Karanika

**Affiliations:** ^1^ Division of Infectious Diseases, Department of Medicine, The Johns Hopkins Hospital, Baltimore, MD, United States; ^2^ Center for Tuberculosis Research, Department of Medicine, Johns Hopkins University School of Medicine, Baltimore, MD, United States; ^3^ Jenner Institute, University of Oxford, Oxford, United Kingdom

**Keywords:** innate immunity, tuberculosis, host-directed adjunct therapies, vaccines, crosstalk with adaptive immunity

*Mycobacterium tuberculosis* (Mtb), the causative agent of tuberculosis (TB), has been a human pathogen since ancient times. TB continues to be one of the world’s deadliest diseases, causing around 1.25 million deaths in 2023 alone (1, 2). A vaccine that offers acceptable pulmonary TB prevention does not yet exist on the market. There is an urgent need for effective preventative TB vaccine development acknowledging the complexity of TB immunity. As it stands, the only widely available vaccine, Bacille Calmette-Guérin (BCG), only prevents TB disease in young children and is ineffective in adolescents and adults (3). TB can be curable with multidrug antibiotic regimens. Still, the lengthy, resource-intensive nature of current treatments poses significant threats to adherence and disease outcomes while simultaneously adding to the growing issue of antibiotic resistance.

Novel immunotherapeutic strategies are long overdue, given that new technologies and insights from other chronic diseases are available today. Innate immune cells are among the first to be affected during TB infection (4), and their molecular interactions with both bacilli and adaptive immune cells are scarcely understood yet critical to advance TB prevention or enhance treatment outcomes. To target it, it is crucial to first deepen our understanding of immune responses in the context of TB prevention, infection, and progression (or non-progression) to active TB disease while elucidating the mechanisms for extensive heterogeneity in immune responses among different hosts. There is immense potential in targeting these cells with creative methodologies.

Zhao et al. present knowledge gaps by innate cell type, outlining areas for future investigation that could shed light on potential Host-Directed Therapy (HDT) targets. HDT targets the host immune response rather than the pathogen and can overcome issues such as drug resistance. Still, innate immune cells’ physiology is more complicated than that since they can be a double-edged sword when infected with Mtb. Although neutrophil degranulation is meant to target the offending pathogen, this process may destroy neighboring cells and cause tissue dissolution (5, 6). Mtb can skew macrophages towards metabolic states that permit bacterial growth and encourage chronic infection, yet precise mechanisms of macrophage plasticity and subset establishment in TB require further study (7). Dendritic cell differentiation is evident to be affected by Mtb since specific Mtb antigens induce altered differentiation, suppress maturation, and increase the expression of their inhibitory molecules (8–10), leading to reduced function. Lastly, Zhao et al. note that natural killer cells are suppressed during Mtb infection, but this process is another area where mechanistic knowledge is scarce. This review also highlights the importance of considering comorbidities and TB risk factors such as diabetes mellitus when studying how differences in host innate immunity contribute to disease outcomes. This is useful not only for insights into molecular mechanisms that make specific populations more susceptible to TB but also for designing therapies specific for certain at-risk populations.

On the other hand, Maceiras et al. focus on how bacterial heterogeneity induces different immune responses. Using a hypervirulent strain of Mtb, this study shows that virulence factors impact host responses by eliciting emergency myelopoiesis with an increased output of myeloid cells early following infection in animals. Still, monocytes or macrophages do not acquire features of trained immunity, and the mechanism through which Mtb inhibits this response requires further study. A purely innate immune model may be useful in studying such mechanisms. Because insects lack adaptive immunity, Nieto Ramirez et al. propose *Galleria mellonella* as a valuable model for studying innate responses to Mtb infection. Exploring such immunologically restricted models could improve our understanding of innate and adaptive responses and the interpretation of findings in murine and non-human primate models, which are widely used for their ability to mimic human TB immunity and pathology.

Mechanistic knowledge of innate immunity in TB has led to the development of novel preventative vaccine strategies. New delivery systems, such as the *Bordetella pertussis* adenylate cyclase (CyaA) toxoid, have proven effective in expanding cell populations and are important for eliminating infected innate immune cells (Badami et al.). Additionally, novel therapeutic strategies have shown promise. One study demonstrated that post-exposure intranasal immunization with nanoparticle and spore-based subunit vaccines boosted both pulmonary and systemic pre-existing Mtb-specific immune responses in mice. Importantly, cells from individuals with latent infection had strong responses to these vaccines, supporting their potential for post-exposure vaccination (Vergara et al.). Antibody-drug conjugates (ADCs), previously explored as cancer therapies, are now being investigated as adjunctive therapies and have been proven to lower bacterial burden in murine models when combined with standard drug-sensitive TB regimen. Wang et al. show that targeting CCRL2 with an ADC, a host protein essential for innate immune cell migration and associated with inflammation, reduces bacterial burden and lung pathology compared with standard treatment alone. This work emphasizes that creative treatment methods already in use for other chronic diseases could prove helpful in managing TB.

In short, novel approaches to prevent and treat Mtb will provide relief from one of the top infectious killers worldwide. Understanding the mechanisms through which innate immune cells promote mycobacterial survival through immune surveillance escape, fail to contain the infection, and interact with adaptive immune cells will open the doors to various new preventative and therapeutic targets. New models are needed that more accurately represent the heterogenous innate responses and their complicated interactions, as well as how those influence the adaptive responses at the early stages of infection. Modulating harmful host responses or boosting beneficial ones is a promising strategy to mitigate risks posed by the limitations of antibiotic therapies. The still poorly understood role of the innate immune cell is central to the development of effective novel preventative measures and therapeutics.
